# Human Mesenchymal Stromal Cell Secretome Promotes the Immunoregulatory Phenotype and Phagocytosis Activity in Human Macrophages

**DOI:** 10.3390/cells9092142

**Published:** 2020-09-22

**Authors:** Minna Holopainen, Ulla Impola, Petri Lehenkari, Saara Laitinen, Erja Kerkelä

**Affiliations:** 1 Finnish Red Cross Blood Service, FI-00310 Helsinki, Finland; ulla.impola@bloodservice.fi (U.I.); saara.laitinen@bloodservice.fi (S.L.); erja.kerkela@bloodservice.fi (E.K.); 2 Molecular and Integrative Biosciences Research Programme, Faculty of Biological and Environmental Sciences, University of Helsinki, FI-00014 Helsinki, Finland; 3 Department of Anatomy and Surgery, Institute of Translational Medicine, University of Oulu and Clinical Research Centre, FI-90014 Oulu, Finland; petri.lehenkari@oulu.fi

**Keywords:** cell therapy, immunomodulation, polyunsaturated fatty acid, CD206, phagocytosis

## Abstract

Human mesenchymal stromal/stem cells (hMSCs) show great promise in cell therapy due to their immunomodulatory properties. The overall immunomodulatory response of hMSCs resembles the resolution of inflammation, in which lipid mediators and regulatory macrophages (Mregs) play key roles. We investigated the effect of hMSC cell-cell contact and secretome on macrophages polarized and activated toward Mreg phenotype. Moreover, we studied the effect of supplemented polyunsaturated fatty acids (PUFAs): docosahexaenoic acid (DHA) and arachidonic acid, the precursors of lipid mediators, on hMSC immunomodulation. Our results show that unlike hMSC cell-cell contact, the hMSC secretome markedly increased the CD206 expression in both Mreg-polarized and Mreg-activated macrophages. Moreover, the secretome enhanced the expression of programmed death-ligand 1 on Mreg-polarized macrophages and Mer receptor tyrosine kinase on Mreg-activated macrophages. Remarkably, these changes were translated into improved *Candida albicans* phagocytosis activity of macrophages. Taken together, these results demonstrate that the hMSC secretome promotes the immunoregulatory and proresolving phenotype of Mregs. Intriguingly, DHA supplementation to hMSCs resulted in a more potentiated immunomodulation with increased CD163 expression and decreased gene expression of matrix metalloproteinase 2 in Mreg-polarized macrophages. These findings highlight the potential of PUFA supplementations as an easy and safe method to improve the hMSC therapeutic potential.

## 1. Introduction

Mesenchymal stromal/stem cells (MSCs) show great promise in cell therapy, such as in the treatment of graft-versus-host disease [1,2] and Crohn’s disease [3,4]. MSCs have diverse immunomodulatory effects, which are mediated via cell-cell contact and secreted paracrine factors, such as extracellular vesicles (EVs), tryptophan-degrading enzyme indoleamine-2,3-dioxygenase and lipid mediator prostaglandin E_2_ (PGE_2_) [5,6,7]. MSCs are able to, e.g., inhibit the proliferation of T cells and promote the generation of regulatory T cells [5,8]. Moreover, MSCs polarize macrophages toward a more anti-inflammatory phenotype by increasing the expression of multiple cell surface markers, such as CD206, and by enhancing their phagocytosis activity [9,10,11,12].

A tight classification of macrophages into different subtypes is redundant due to their plastic and rapidly changing phenotype giving rise to a heterogeneous population [13]. Yet for simplicity, macrophages are typically classified into classically activated, proinflammatory M1 phenotype and to wound healing, anti-inflammatory M2 phenotype. Regulatory macrophages (Mregs) represent an immunoregulatory phenotype that produce anti-inflammatory cytokines, such as interleukin (IL)-10 and transforming growth factor β1 (TGF-β1), potently suppress T-cell function and promote regulatory T-cell phenotype [14,15]. Interestingly, Mregs are also investigated as a potential adjunct therapy in renal transplantations (clinicaltrials.gov: NCT02085629). Although the effects of MSCs on monocytes and type M1 and M2 macrophages have been intensively studied, less is known about the effects of MSCs on Mregs. In our previous study, we observed that human bone marrow-derived MSCs (hBMSCs) and hBMSC-derived EVs (hBMSC-EVs) enhanced the anti-inflammatory phenotype of Mregs [16]. Both hBMSC cell-cell contact and EVs decreased the production of IL-23 and IL-22, which are up-regulated in inflammation and promote T helper 17 cell maintenance and proliferation, respectively. The hBMSCs and EVs also increased the production of PGE_2_ [16], which is an essential lipid mediator in MSC function and induces the MSC-mediated skewing of macrophages toward an anti-inflammatory phenotype [11,17].

EVs are small (majority < 300 nm) lipid-bilayered particles secreted by cells through exocytosis and membrane budding. EVs carry intracellular messages by transporting lipids, proteins, nucleic acids, carbohydrates or their metabolites and can mediate immunological effects [18]. Intriguingly, MSC-EVs are able to mediate the therapeutic response of MSCs and have been investigated in various in vivo models, such as acute kidney injury [19], stroke [20,21] and sepsis [22]. Thus, MSC-EVs have emerged as a cell-free therapeutic option for MSCs.

The immunomodulatory response of MSCs resembles the resolution of inflammation, the active dampening phase of inflammation [23]. Lipid mediators, especially the specialized proresolving mediators (SPMs) promote the resolution of inflammation [24] by, e.g., reducing neutrophil trafficking, increasing macrophage polarization toward anti-inflammatory phenotype and macrophage efferocytosis of apoptotic neutrophils. Polyunsaturated fatty acids (PUFAs), such as *n*-3 docosahexaenoic acid (DHA), eicosapentaenoic acid (EPA) and *n*-6 arachidonic acid (AA) are precursors to lipid mediators [25]. DHA is a precursor to resolution-phase SPMs such as D-series resolvins, maresins and protectins and EPA to E-series resolvins. AA is a precursor to proresolving lipoxins, but also for prostaglandins (PGs), thromboxanes and leukotrienes with mainly proinflammatory functions.

We have previously demonstrated that the phospholipid, fatty acid and, importantly, lipid mediator profiles of hBMSCs can be modified with the supplementation of PUFAs [26,27]. hBMSCs cannot efficiently synthetize these long-chained PUFAs from *n*-6 and *n*-3 precursors rendering the PUFA supplementation into the culture medium essential to ensure a sufficient level of precursors for lipid mediator biosynthesis [26]. Interestingly, we also observed that PUFA supplementation to hBMSCs caused the subsequent remodeling of the phospholipid membrane of hBMSC-EVs [27]. Furthermore, EPA and DHA supplementation to murine MSCs increases their immunomodulatory capacity in allergic asthma [28] and sepsis [29,30] models, highlighting the importance of PUFA modifications on MSC immunomodulation.

In this study, we investigated the effect of hBMSC cell-cell contact and secretome on polarized and activated Mregs. Moreover, we investigated the immunomodulatory effect of hBMSCs on Mregs after DHA or AA supplementations, which alter the downstream lipid mediator profile of hBMSCs. Our results demonstrate that the hBMSC secretome skewed macrophages toward a more anti-inflammatory and proresolving phenotype. This effect was even more pronounced by the secretome of DHA-modified hBMSCs. Strikingly, we show for the first time that the hBMSC secretome enhanced the *Candida albicans* phagocytosis activity of macrophages by increasing the CD206 expression.

## 2. Materials and Methods

### 2.1. hBMSC Culture and PUFA Supplementation

The patient protocols of the hBMSC isolation were approved by the Ethical Committee of Northern Ostrobothnia Hospital District (ethical approval number: Oulu University hospital EETTMK 21/2011). The hBMSCs were collected from upper femur metaphysis of adult patients after receiving a written informed consent and characterized as described previously [31]. The cells have been characterized according to the guidelines of the International Society of Cell & Gene Therapy [32]. The cells expressed typical MSC markers and lacked the expression of hematopoietic stem cell markers, and the differentiation toward osteoblasts and adipocytes was also tested (data not shown). hBMSCs derived from three donors were inspected in this study.

The cells were thawed, cultured [27] and PUFAs supplemented [26] as described previously. In brief, the hBMSCs at confluence of 80–90% were supplemented with ethanol (purity ≥99.5%, Altia Industrial, Rajamäki, Finland) as a control, DHA or AA (Cayman Chemical, Ann Arbor, MI, USA) as bovine serum albumin (BSA, Merck, Darmstadt, Germany) conjugates. Prior to the PUFA supplementation, the medium was changed to medium containing only 5% fetal bovine serum (FBS, Thermo Fisher Scientific, Waltham, MA, USA) in contrast to 10% FBS in the proliferation medium. The PUFAs dissolved in ethanol were added into 1.5 mM BSA-Dulbecco’s phosphate buffered saline (DPBS, Thermo Fisher Scientific) solution, vortexed and immediately added to the cell culture medium. The final PUFA concentration supplemented to the cells was 50 µM. After 24 h, hBMSCs were detached and 50,000 cells/well were added into Mreg polarization assay.

### 2.2. Mreg Polarization Assay

**Figure 1:**
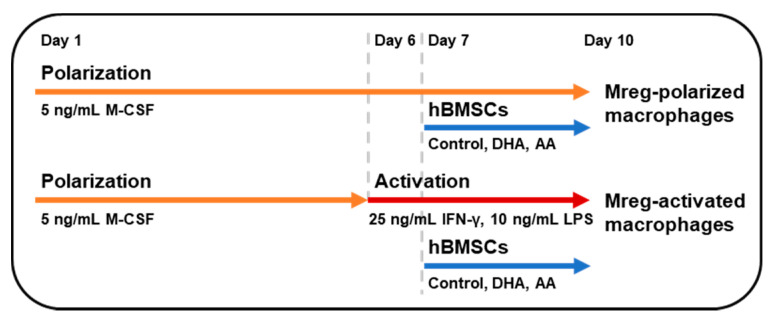
The layout of macrophage polarization assay. Mreg, regulatory macrophage; hBMSC, human bone marrow-derived mesenchymal stromal cell; DHA, docosahexaenoic acid; AA, arachidonic acid; M-CSF, macrophage colony-stimulating factor; IFN, interferon; LPS, lipopolysaccharide.

The use of anonymized peripheral blood mononuclear cells (PBMCs) from blood donors in research is in accordance with the rules of the Finnish Supervisory Authority for Welfare and Health (Valvira, Helsinki, Finland). The layout of the assay is described in Figure 1 and macrophages derived from six different donors were used in the assay. The Mregs were cultured as described in [16] with certain changes. In brief, 2 × 10^6^–4 × 10^6^ PBMCs were plated on 12-well plates (Corning™ Costar™ flat bottom, Thermo Fisher Scientific), incubated for 1–2 h and washed with DPBS. The attached monocytes were incubated in 1.5 mL RPMI-1640 medium (Thermo Fisher Scientific) with 10% FBS (Merck), GlutaMAX™ supplement (Thermo Fisher Scientific) and 5 ng/mL macrophage colony-stimulating factor (M-CSF, PromoCell, Heidelberg, Germany) for 6 days at 37 °C, 5% CO_2_. This medium is referred as polarization medium and macrophages obtained in these conditions are referred as Mreg-polarized macrophages from here onwards. At day 6, the medium was changed into the polarization or activation medium [polarization medium with 25 ng/mL interferon (IFN)-γ and 10 ng/mL lipopolysaccharide (LPS, both from Merck)]. Macrophages cultured in the activation medium are referred as Mreg-activated macrophages. The next day, 50,000 control-hBMSCs, DHA-hBMSCs or AA-hBMSCs were added to the bottom of wells (referred as hBMSC cell-cell contact) or to inserts (Corning™ Transwell™, pore size 0.4 µm, Thermo Fisher Scientific) (referred as the hBMSC secretome).

At day 10, the medium was centrifuged at 300× *g* for 15 min and the supernatant was snap frozen and stored at −80 °C. The cells were washed with DPBS and either detached for flow cytometry with 0.75 µL 4 °C Macrophage Detachment Solution DFX (PromoCell) or scraped into 600 µL RLT lysis buffer (Qiagen, Hilden, Germany) for quantitative polymerase chain reaction (QPCR). The RLT samples were snap frozen and stored at −80 °C.

### 2.3. Determination of Cytokine Production

The medium samples were thawed on ice and analyzed with human tumor necrosis factor (TNF)-α, IL-10 and IL-23 DuoSet enzyme-linked immunosorbent assays (ELISA) (all from R&D Systems, Minneapolis, MN, USA) according to the manufacturer’s protocol. The absorbance was measured with CLARIOstar^®^ microplate reader (BMG Labtech, Ortenberg, Germany).

### 2.4. Macrophage Phenotyping with Real-Time Quantitative PCR

Only the macrophages with hBMSC secretome samples (hBMSCs cultured on an insert) were analyzed with real-time QPCR, because cell-cell contact samples included both macrophages and hBMSCs. The RNA was extracted using RNeasy Mini Kit (Qiagen) according to the manufacturer’s protocol. The concentration and purity of RNA was measured with NanoDrop ND-1000 spectrophotometer (Thermo Fisher Scientific). RNA was converted to complementary DNA with High Capacity cDNA RT Kit (Thermo Fisher Scientific). The gene expression was analyzed with real-time QPCR (CFX96™ Real-Time Systems and C1000™ Thermal Cycler, Bio-Rad, Hercules, CA, USA) using TaqMan^®^ Gene Expression assays and TaqMan^®^ Universal Master Mix II (Thermo Fisher Scientific). The following genes were analyzed: *TGFB1* (ID: Hs00998133_m1), *MMP2* (ID: Hs01548727_m1), *DHRS9* (ID: Hs00608375_m1), *STAT3* (ID: Hs00374280_m1) and *STAT1* (ID: Hs01013996_m1). *HPRT1* was used as the reference gene. Samples were analyzed as duplicates and the results were analyzed with CFX Manager™ 3.0 (Bio-Rad) and with the 2^−ΔΔCt^ method using *HPRT1* as the reference gene [33]. The relative gene expression levels are expressed as log2 fold change relative to the Mreg-polarized or Mreg-activated macrophages cultured without hBMSCs.

### 2.5. Macrophage Phenotyping with Flow Cytometry

The antibody staining was performed as described in Hyvärinen et al. (2018) using anti-human antibodies PE-CF594-CD86 (clone 2331 FUN-1), APC-CD206 (clone 19.2), BV421-CD163 (clone GHI/61), PerCP-Cy™5.5-CD90 (clone 5E10) (from BD Biosciences, San Diego, CA, USA), FITC-HLA-DR (clone L243), BV 510™-CD274 (PD-L1, B7-H1, clone 29E.2A3), PE/Cy7-CD120b (TNFR2, clone 3G7A02) (from BioLegend, San Diego, CA, USA) and PE-MERTK (clone HMER5DS, Thermo Fisher Scientific). Cell viability was determined with LIVE/DEAD™ Fixable Near-IR Dead Cell Stain Kit (Thermo Fisher Scientific). Data was acquired with BD FACSAria IIU (BD Biosciences) flow cytometer using FACSDiva™ (v8.0.1, BD Biosciences) and analyzed with FlowJo^®^ (v10, BD Biosciences). Gating strategy and doublet discrimination is depicted in Figure S1. Fluorescence positive cells were determined by using isotype controls and CD90 positive cells were excluded from the analysis (Figure S1). The results are presented as median fluorescence intensity (MFI) and frequency of positive cells and as their log2 fold change values relative to the Mreg-polarized or Mreg-activated macrophages cultured without hBMSCs.

### 2.6. Yeast Heat-Inactivation and CFSE-Staining

Lyophilized *C. albicans* pellets (ATCC^®^ 10231™, Microbiologics, Saint Cloud, MN, USA) were dissolved in 1 mL NaCl Peptone Broth solution (Merck) for 30 min at 37 °C according to the manufacturer’s protocol. The yeast solution was incubated for 1 h in 80 °C to kill the cells [34]. Yeast solution (1 × 10^7^ cells/mL) was stained with 2 µM carboxyfluorescein succinimidyl ester (CFSE, Thermo Fisher Scientific) for 15 min at 37 °C. The stained yeast cells were washed with RPMI 1640 and centrifuged at 1000× *g* for 5 min. The pellet was suspended with polarization medium at concentration of 5 × 10^6^ cells/mL and filtered. The CFSE-stained *C. albicans* were stored o/n at 4 °C. The staining was verified with imaging flow cytometry (Amnis^®^ ImageStream^®^X Mark II, Luminex Corporation, Austin, TX, USA). The viability of heat-killed yeast was tested by the absence of growth on agar plates after 48 h incubation at 37 °C.

### 2.7. Phagocytosis Assay with Imaging Flow Cytometry

Polarized macrophages were cultured with control-hBMSCs on inserts as described above. Macrophages derived from five different donors were employed in the phagocytosis assay. On day 10, the hBMSC inserts were removed and the medium was replaced with 1 mL polarization medium containing heat-killed CFSE-stained *C. albicans* at low (5 × 10^5^ cells/well) or high (1.25 × 10^6^ cells/well) concentration. The plates were centrifuged at 30× *g* for 1 min to synchronize the phagocytosis in all wells and incubated for 45 min at 37 °C, 5% CO_2_. The cells were washed with DPBS, detached, stained with PE-CF594-CD86 and APC-CD206 and analyzed with imaging flow cytometry.

Amnis^®^ ImageStream^®^X Mark II 12-channel imaging flow cytometer with the software INSPIRE^®^ (Luminex Corporation) was used for data collection and analysis. Acquisition settings were as follows: Excitation lasers 405 (off), 488 (15 mW), 642 (150 mW) and 785 (6.75 mW) were applied for the excitation of fluorochromes and laser Channels (Ch) 01 and Ch09 (bright field, BF), Ch06 (scattering channel, SSC), plus fluorescence channels Ch02, Ch04 and Ch11 were activated for signal detection.

All acquisition settings were the same in all experiments. Single cells were separated from debris and aggregates in the BF channel using the IDEAS features aspect ratio and area. Samples were acquired at 60× magnification with low flow rate/high sensitivity. At least 2000 events of gated single cells for each sample were collected. Single color controls were used to create a compensation matrix. Unlabeled cells and isotype control samples were used to determine the auto fluorescence and the non-specific background.

Compensated data files were analyzed using algorithms available in the IDEAS^®^ analysis software (v6.2.188.0). Positive events were gated based on cell morphology and the intensity values of each fluorescence channel and cell BF images (Figure S2). Gating and compensation values were used as analysis template for all experimental files. This batch processing of all files assured the comparisons of each experiment with the other.

### 2.8. Statistical Analysis

Non-parametric tests were performed due to the non-normal distribution of parameters. The results are expressed as medians with interquartile ranges (IQR). Pairwise statistical testing was conducted with Wilcoxon signed rank test. Groupwise statistical testing was conducted with Kruskal-Wallis rank sum test and post hoc pairwise comparisons using Dunn’s test for multiple comparisons with Mreg-polarized or Mreg-activated macrophages cultured without hBMSCs. All statistical tests were conducted with R version 3.5.1 and the PMCMR package [35,36] and *p*-value < 0.05 was considered significant.

## 3. Results

### 3.1. Phenotype of Polarized and Activated Macrophages

The phenotypes of macrophages were determined by flow cytometry using markers for T-cell activation costimulatory molecule CD86, major histocompatibility complex class II cell surface receptor human leukocyte antigen (HLA)-DR, mannose receptor CD206, scavenger receptor CD163, programmed death-ligand 1 (PD-L1, also known as CD274), tumor necrosis factor receptor 2 (TNFR2) and Mer receptor tyrosine kinase (MerTK) (Tables S1 and S2). hBMSCs were excluded using CD90 labeling and were detectable only in the samples with hBMSC cell-cell contact (Figure S1). The median frequency of dead cells of Mreg-polarized macrophages was 1.6% (IQR 2.5) and 1.9% (IQR 5.4) for Mreg-activated macrophages.

The phenotype of Mreg-polarized macrophages is shown in Tables S1 and S2. We observed considerable donor-specific variation in response to Mreg polarization conditions and subsequently in the expression of phenotype markers between different buffy coat donors. Approximately, 78% of these macrophages were positive for CD86, 99.7% for HLA-DR, 35% for CD206, 6% for CD163, 59% for PD-L1 and <1% for both TNFR2 and MerTK. The phenotype of Mreg-activated macrophages (mature Mregs) was similar to that of Mreg-polarized macrophages (Tables S1 and S2). Approximately 97% of the activated macrophages were positive for CD86, 99.9% for HLA-DR, 23% for CD206, <1% for CD163, 92% for PD-L1, negative for TNFR2 and <1% for MerTK. In general, the expression levels of phenotype markers were similar between the two macrophage types, but the Mreg-activated macrophages had a higher frequency of CD86+ (pairwise Wilcoxon signed rank test, *p* = 0.031) and PD-L1+ cells (*p* = 0.031) than Mreg-polarized macrophages.

Both macrophage types produced TNF-α and IL-10 (Table 1). The production of TNF-α increased in Mreg-activated macrophages compared with Mreg-polarized macrophages (pairwise Wilcoxon signed rank test, *p* = 0.031) while the production of IL-10 remained at similar levels. The production of IL-23 was negligible. The donor-specific variation was high in the cytokine production.

Both Mreg-polarized and Mreg-activated macrophages expressed TGF-β1, matrix metalloproteinase (MMP)-2, human Mreg marker dehydrogenase/reductase 9 (DHRS9) [15], signal transducer and activator of transcription (STAT)3 and STAT1, determined with QPCR. There were no statistically significant differences in gene expression between the two macrophage types (not shown). Expression was, again, variable depending on the donor.

### 3.2. hBMSC Secretome Skews Mreg-Polarized and Mreg-Activated Macrophages toward an Anti-Inflammatory and Proresolving Phenotype

**Figure 2:**
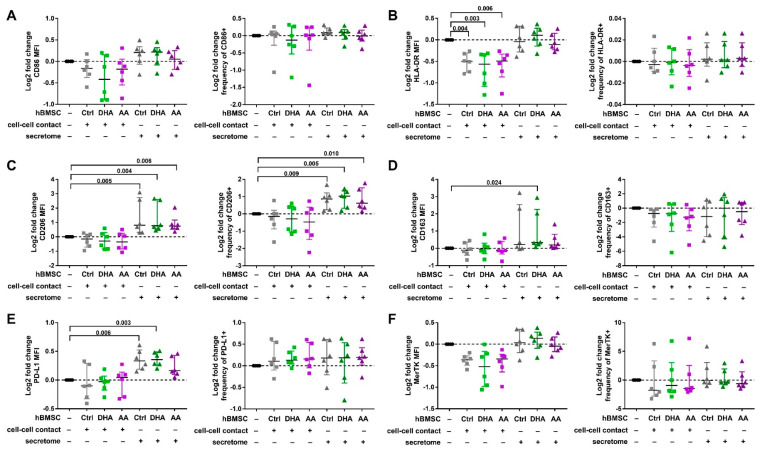
The effect of hBMSC cell-cell contact and secretome on the cell surface the phenotype of Mreg-polarized macrophages. The median fluorescence intensities (MFI, on the left in each panel) and frequencies of positive cells (on the right in each panel) were determined with flow cytometry investigating the expression of (**A**) CD86, (**B**) HLA-DR, (**C**) CD206, (**D**) CD163, (**E**) PD-L1 and (**F**) MerTK. The effect of hBMSCs is visualized with log2 fold changes calculated against macrophages cultured without hBMSCs (represented by the zero line) for each individual donor. The differences among groups were determined with Kruskal-Wallis rank sum test and post hoc using Dunn’s test (the latter presented in the figures). The results are expressed as log2 fold changes as medians with interquartile ranges; *n* = 6 biological replicates. Ctrl, control; DHA, docosahexaenoic acid; AA, arachidonic acid.

The phenotype of both Mreg-polarized and Mreg-activated macrophages was modified especially by the hBMSCs secretome. The effect of hBMSC cell-cell contact and secretome on the cell surface protein expression of Mreg-polarized macrophages is shown in Figure 2 and Tables S1 and S2. Strikingly, the hBMSC secretome increased the log2 fold change of both CD206 MFI (Kruskal-Wallis test with all hBMSC conditions, *p* < 0.001; post hoc test *p*-values are described in Figure 2) and frequency of CD206+ cells (*p* = 0.002) regardless of the PUFA supplementation. The secretome of control- and DHA-hBMSCs elevated also the log2 fold change of PD-L1 MFI (*p* < 0.001) and DHA-hBMSCs the log2 fold change of CD163 MFI (*p* = 0.039), but the effect was donor-dependent. Interestingly, hBMSC cell-cell contact with all PUFA modifications decreased the log2 fold change of HLA-DR MFI (*p* = 0.001).

**Figure 3:**
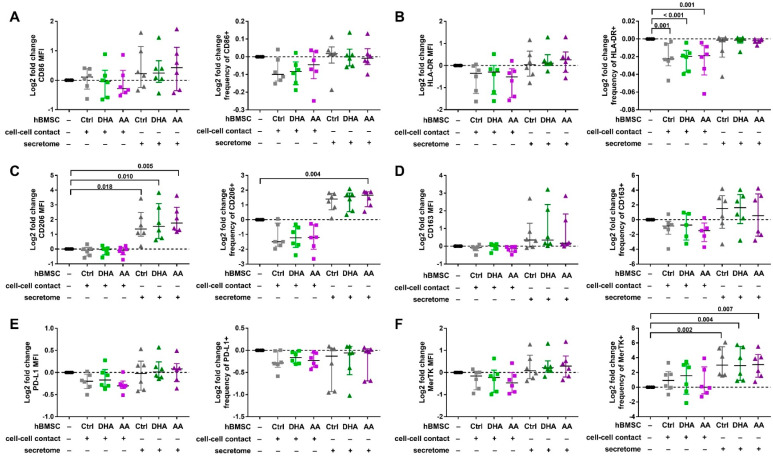
The effect of hBMSC cell-cell contact and secretome on the cell surface the phenotype of Mreg-activated macrophages. The median fluorescence intensities (MFI, on the left in each panel) and frequencies of positive cells (on the right in each panel) were determined with flow cytometry investigating the expression of (**A**) CD86, (**B**) HLA-DR, (**C**) CD206, (**D**) CD163, (**E**) PD-L1 and (**F**) MerTK. The effect of hBMSCs is visualized with log2 fold changes calculated against macrophages cultured without hBMSCs (represented by the zero line) for each individual donor. The differences among groups were determined with Kruskal-Wallis test and post hoc using Dunn’s test. The results are expressed as log2 fold changes as medians with interquartile ranges; *n* = 6 biological replicates. Ctrl, control; DHA, docosahexaenoic acid; AA, arachidonic acid.

**Figure 4:**
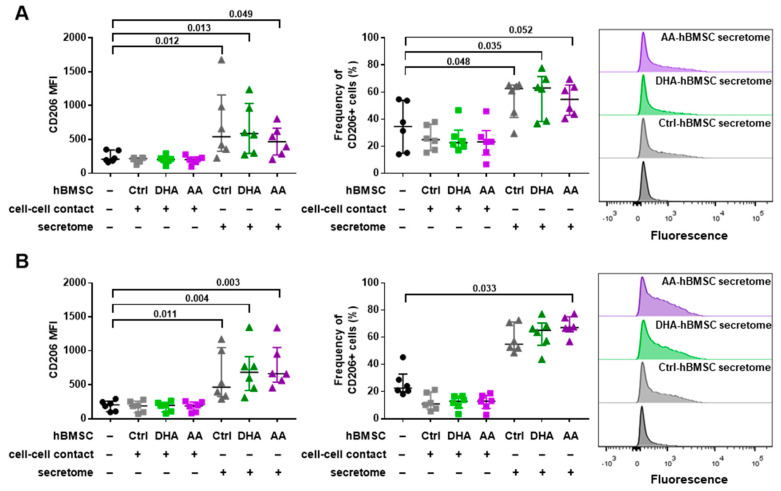
The secretome of hBMSCs increases the expression of CD206. (**A**) The upper panel describes the results of Mreg-polarized macrophages and (**B**) the lower panel the results of Mreg-activated macrophages. The median fluorescence intensities (MFI, left panel) and frequencies of positive cells (middle panel) were determined with flow cytometry. The representative histograms are presented on the right. The differences among groups were determined with Kruskal-Wallis rank sum test and post hoc using Dunn’s test. These results are also described as log2 fold changes in Figure 2 and Figure 3. The results are expressed as medians with interquartile ranges; *n* = 6 biological replicates. Ctrl, control; DHA, docosahexaenoic acid; AA, arachidonic acid.

The effect of hBMSC cell-cell contact and secretome on the cell surface protein expression of Mreg-activated macrophages is shown in Figure 3 and Tables S1 and S2. Similar to Mreg-polarized macrophages, the log2 fold change of CD206 MFI and the frequency of CD206+ cells increased in the Mreg-activated macrophages by the secretome of hBMSCs with all PUFA modifications (Kruskal-Wallis test of all hBMSC conditions *p* < 0.001; post hoc test *p*-values are described in Figure 3) and with AA supplementation (*p* < 0.001), respectively. Moreover, the secretome elevated the log2 fold change frequency of MerTK+ cells regardless of the PUFA supplementation (*p* = 0.014). The expression was still low (<15% positive cells) and the increase was variable between donors. The cell-cell contact had an effect on only the log2 fold change frequency of HLA-DR+ cells, which was slightly decreased regardless of the PUFA modification (*p* < 0.001). The most drastic change in both Mreg-polarized and Mreg-activated macrophages was the elevated CD206 expression, which is depicted in Figure 4 (presenting the CD206 data in more detail without the log2 fold change values).

**Figure 5:**
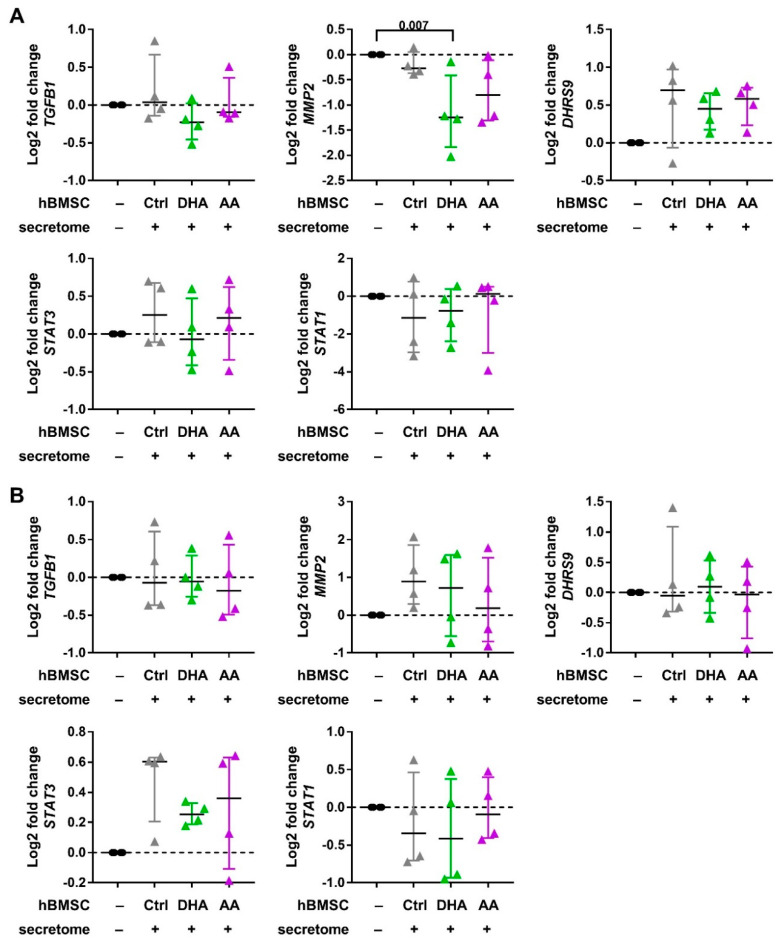
The effect of hBMSC secretome on the gene expression of Mreg-polarized and Mreg-activated macrophages. The gene expression of macrophage phenotype markers was investigated with QPCR. The effect of hBMSCs on (**A**) Mreg-polarized and (**B**) Mreg-activated macrophages is visualized with log2 fold changes calculated against macrophages cultured without hBMSCs (represented by the zero line) for each individual donor. The differences among groups were determined with Kruskal-Wallis rank sum test and post hoc using Dunn’s test. The results are expressed as medians with interquartile ranges; *n* = 4 biological replicates. Ctrl, control; DHA, docosahexaenoic acid; AA, arachidonic acid.

The hBMSCs had no effect on the cytokine production in this experimental setting (Table 1). In the coculture with Mreg-polarized macrophages, the production of IL-23 increased in by the cell-cell contact of control-hBMSCs (Kruskal-Wallis test *p* = 0.015; post hoc Dunn’s test, *p* = 0.002); however, the production of IL-23 was at the detection limit and this result should be interpreted with caution. The expression of most genes investigated remained unaltered by the hBMSC secretome (Figure 5). Nevertheless, DHA-hBMSCs resulted in a decreased MMP-2 gene expression in the Mreg-polarized macrophages (Kruskal-Wallis test *p* = 0.031; post hoc Dunn’s test, *p* = 0.007) and AA-hBMSCs caused a similar trend. There was also a trend of increased gene expression of the Mreg marker DHRS9 in DHA and AA-hBMSCs coculture.

### 3.3. Phagocytosis Assay

**Figure 6:**
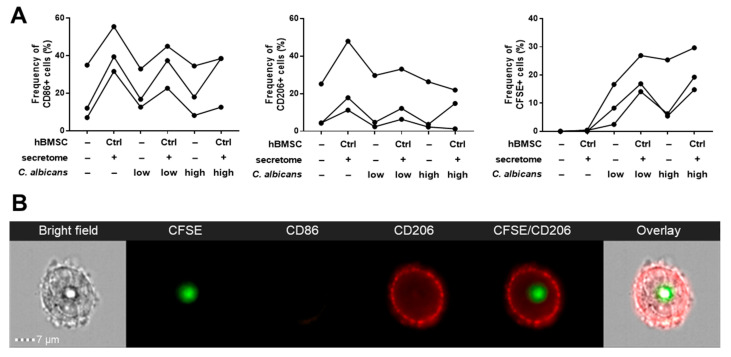
hBMSC secretome improves the *C. albicans* phagocytosis activity of Mreg-polarized macrophages in a CD206-mediated manner. (**A**) The frequency of CD86 and CD206 positive cells and the phagocytosis of CFSE-stained *C. albicans* were determined with imaging flow cytometry, *n* = 3 biological replicates. (**B**) A representative imaging flow cytometry image of a macrophage that has phagocytosed *C. albicans*. Low *C. albicans* concentration, 5 × 10^5^ cells/well; high *C. albicans* concentration, 1.25 × 10^6^ cells/well; CFSE, CFSE-stained *C. albicans*.

The *C. albicans* phagocytosis activity of Mreg-polarized macrophages was assessed with/without hBMSCs secretome and the results analyzed with imaging flow cytometry (Figure 6). The macrophages elicited a donor-dependent CD86 and CD206 expression. In contrast to the previous results, hBMSC secretome did not increase the CD206 expression in all of the macrophages derived from different PBMC donors (non-responders *n* = 2, Figure S3). However, in three cases, the CD206 increased by 1.9 to 4.2 fold when compared with the non-responders, which elicited a 0.7 to 0.8-fold decrease in the CD206 expression. The results of the CD206 responders are reported in Figure 6. In the responders, the phagocytosis of low concentration (5 × 10^5^ cells/well) *C. albicans* increased by 1.6 to 5.8 fold in the hBMSC secretome group when compared with Mreg-polarized macrophages alone. In general, the levels of ingested *C. albicans* were higher in the high concentration (1.25 × 10^6^ cells/well) group and the phagocytosis increased by 1.2 to 3.1-fold in the hBMSC secretome group when compared with Mreg-polarized macrophages alone.

## 4. Discussion

We investigated the effect of hBMSC cell-cell contact and secretome to the phenotype of immunoregulatory macrophages, which were either polarized (Mreg-polarized) or polarized and activated (Mreg-activated) toward Mreg phenotype. Moreover, we supplemented hBMSCs with PUFAs DHA or AA prior to the macrophage coculture to elucidate if PUFAs induced changes in the immunomodulatory capacity of hBMSCs. The hBMSC secretome increased the expression of anti-inflammatory and proresolving phenotype markers in both macrophage types with all hBMSC modifications (control, DHA and AA). In particular, we observed a substantial increase in the CD206 expression. Furthermore, the hBMSC secretome increased donor-dependently the phagocytosis activity of Mreg-polarized macrophages in *C. albicans* phagocytosis assay, a result which was associated with the increased CD206 expression. Intriguingly, the DHA-supplemented hBMSCs induced the most prominent anti-inflammatory changes in Mreg-polarized macrophages while AA supplementation had only a slight effect.

Our aim was to study macrophages both in a more naïve stage (Mreg-polarized macrophages) and as mature Mregs (Mreg-activated macrophages) [37] in order to elucidate the effect of hBMSCs on macrophages at different polarization stages. The phenotypes of these two macrophage types were similar, but the Mreg-activated macrophages manifested a more classically activated phenotype with increased expression of CD86 and PD-L1. Moreover, the Mreg-activated macrophages produced more TNF-α than Mreg-polarized macrophages as expected due to Toll-like receptor engagement by LPS.

Previously, we demonstrated that hBMSC cell-cell contact and hBMSC-EVs enhanced the anti-inflammatory properties of mature Mregs by decreasing the production of IL-23 and IL-22 and increasing the PGE_2_ production [16]. In the current study, we did not detect a decrease in the IL-23 production, which was very low when measured with ELISA (previously analyzed with ProcartaPlex Immunoassay), but we observed other anti-inflammatory effects of hBMSCs on these immunoregulatory macrophages. Additionally, the experimental setup and the markers investigated of these two studies were slightly different.

The hBMSC secretome enhanced the anti-inflammatory properties of both Mreg-polarized and Mreg-activated macrophages especially by increasing the CD206 expression. CD206, also known as mannose receptor, is a pattern recognition receptor, which binds to the glycan structures on microbes [38]. Previous studies have shown that human MSC cell-cell contact or secretome can increase the expression of CD206 and overall anti-inflammatory properties of monocytes or M1 macrophages [9,11]. Moreover, human MSC-derived EVs can increase the CD206 and anti-inflammatory properties of murine macrophages [39]. The secretome consists of secreted cytokines, lipid mediators and other molecules and also includes EVs. Thus, our results with macrophages polarized toward Mregs are in agreement with previous studies investigating monocytes or other macrophage subtypes and CD206 expression. In our preceding study with Mregs, we did not observe an increase in the CD206 expression with hBMSC-EV addition [16]; however, the experimental settings of our two studies are not directly comparable. Previously, the EVs added to Mregs were derived from unstimulated hBMSCs and given in two doses. In the current study, the hBMSCs produced the EVs and additional soluble factors constantly in a stimulated environment with macrophage coculture.

Next, we investigated if the increased CD206 expression translated to an increased phagocytosis activity of yeast *C. albicans* by Mreg-polarized macrophages in the presence of hBMSC secretome. The cell surface of *C. albicans* is covered in terminal mannose residues that are recognized by CD206 of macrophages [38,40]. Interestingly, when the hBMSC secretome increased the CD206 expression in the responder macrophages, the phagocytosis of *C. albicans* was also increased indicating an association between these two hBMSC secretome-mediated phenomena. The effect was PBMC donor-dependent, because the donors responded to the polarization and hBMSC secretome differently. Both human and murine MSCs have been shown to induce the phagocytosis of macrophages in various assays [10,12,41] but to our knowledge this is the first study showing that MSCs induce the phagocytosis of *C. albicans*. One of the key aspects in the resolution of inflammation is the clearance of pathogens and apoptotic cells via phagocytosis and efferocytosis [24]. Phagocytosing macrophages become more prevalent during resolution of inflammation when the cell debris and microbes are cleared away in order to achieve homeostasis [24] emphasizing the ability of hBMSC secretome to promote the proresolving phenotype of macrophages.

In addition to CD206, the PD-L1 expression increased by the hBMSC secretome in Mreg-polarized macrophages. When PD-L1 binds to its receptor, a co-inhibitory receptor programmed death 1 (PD-1), T-cell activation and proliferation are inhibited and the immune response is attenuated [42]. PD-L1 is expressed on the surface of proresolving macrophages [43], which indicates that the hBMSC secretome skews the macrophage phenotype in an anti-inflammatory direction. Supporting our results, MSCs have been shown to increase the PD-L1 expression in M2-type macrophages [10].

Strikingly, in Mreg-activated macrophages, the hBMSC secretome increased the expression of MerTK, which was generally negative in the cells. The different PBMC donors; however, responded in a different manner. MerTK is a marker of anti-fibrotic M2c macrophages [44], it is important in the clearance of apoptotic cells [45] and induces SPM production in macrophages [46]. Interestingly, EVs from cardiosphere-derived cells are able to increase the MerTK expression of macrophages via the transfer of microRNA-26a [47], which may indicate that the EVs in the hBMSC secretome are mediating the increased MerTK expression. It has been well established that EVs mediate a large proportion of the immunomodulatory effects of MSCs [48]. Although we did not examine the effect of EVs alone, we can hypothesize that observed anti-inflammatory and proresolving effects of hBMSC secretome were at least in part mediated by the EV fraction.

The secretome of hBMSCs had a larger impact on the phenotype of macrophages than the hBMSC cell-cell contact. Mainly, the cell-cell contact lowered the HLA-DR expression of Mreg-polarized and, to a certain extent, Mreg-activated macrophages. HLA-DR is a proinflammatory marker and is involved in the T-cell activation via antigen presentation. In agreement with our result, human MSCs can diminish the HLA-DR expression of macrophages and monocytes [49,50] indicating that the hBMSC cell-cell contact also rendered the macrophage phenotype more anti-inflammatory.

The PUFA supplementation of hBMSCs prior the coculture with macrophages resulted in a more pronounced anti-inflammatory phenotype of Mregs. In particular, the secretome of DHA-hBMSCs increased the CD163 expression and decreased the gene expression of gelatinase MMP-2 in Mreg-polarized macrophages while control- or AA-hBMSCs had no effect. CD163 is a hemoglobin scavenger receptor inducing an anti-inflammatory response and its increased expression is one of the major changes in the macrophage switch to alternatively activated phenotype [51]. On the other hand, M1 macrophages secrete MMP-2 to induce the degradation of extracellular matrix and recruitment of inflammatory cells to the site of tissue injury [52]. Previous studies have shown that MSCs are able to decrease the gene expression of MMP-2 of macrophages in vitro [53] and in vivo [54] corroborating with our results. The secretome of AA-hBMSCs did not have a significant effect on the PD-L1 expression in Mreg-polarized macrophages while control and DHA-hBMSCs increased this expression. This result hints that AA supplementation could lower the immunosuppressive properties of hBMSCs, because PD-L1 is important in suppressing the T-cell mediated immune response [42]. Contrastingly, the increase in CD206 expression in Mreg-activated macrophages was the most prominent with AA-hBMSC secretome.

Previous in vivo studies have reported that EPA-supplemented murine MSCs had superior therapeutic potential when compared with control MSCs [28,30]. Moreover, DHA-supplemented murine MSCs increased the survival in an in vivo sepsis model when compared with AA-supplemented MSCs [29]. In this study, we observed more prominent anti-inflammatory changes in Mregs with DHA-hBMSCs than with control or AA-hBMSCs; however, the effect was visible in only two phenotype markers. The lack of a substantial effect of PUFA supplementation to hBMSC immunomodulation in this in vitro assay may be due to different reasons. Firstly, the PUFA remodeling of hBMSC membranes takes place relatively quickly, beginning already after 2 h after PUFA supplementation and leading into prominent remodeling after 24 h [27]. In the current in vitro setting, the medium in the 3-day macrophage-hBMSC culture contained 10% FBS. Even though this FBS most likely has modified the cell membranes of hBMSCs and lowered the PUFA content of the membranes, we hypothesized that the initial modifications in the hBMSC membranes would suffice to induce profound changes already at the beginning of the coculture. This hypothesis is supported by the studies, where the EPA- and DHA-supplemented MSCs demonstrated improved therapeutic potential [28,29,30], even though the cell membranes of these cells would most likely be remodeled in vivo.

Additionally, our in vitro setting focused on changes in polarized and activated Mregs and DHA and AA supplementation. Although limited in this experimental setting, PUFA modifications could still have an impact in hBMSC immunomodulatory properties in other immunological settings. Moreover, EPA supplementation has been shown to be beneficial for MSC immunomodulation [28,30]. EPA is the precursor to PGE_3_, which is less proinflammatory than PGE_2_ [55] and could be one of the underlying reasons for the pronounced effect of EPA supplementation to MSC immunomodulation. Thus, the effects of PUFA-modified hBMSCs call for further investigations. If PUFA supplementations of MSCs prove out to be beneficial, these supplementations represent an easy and safe way to improve the therapeutic response of MSCs.

The macrophage assays were conducted with primary human PBMCs from individual donors. We acknowledge the challenges of using primary PBMC-derived macrophages as a model due to their high plasticity and variability in responding to cytokines and activation. However, by employing primary human cells, we achieve a more realistic setting than by the use of cell lines, representing a more physiologically accurate situation and acknowledging the varying responses of the patients in the clinic. Indeed, the magnitude of the response to different assay conditions and subsequently, the phenotypes of macrophages derived from individual donors were variable. The hBMSC secretome induced prominent differences in Mreg-polarized and Mreg-activated macrophages. Some of the changes in phenotype markers, as in CD206, were clear in all individual donors by the hBMSC secretome highlighting the significance of this finding. It is also noteworthy that although some of the effects were small, all of the changes skewed macrophages toward an anti-inflammatory and proresolving direction. Moreover, we investigated the effects of hBMSCs derived from three different donors to multiple buffy coat derived PBMCs, which enhances the robustness of our findings.

To conclude, our results demonstrate that the hBMSC secretome can modify macrophages toward immunoregulatory and proresolving phenotype, especially by increasing CD206, PD-L1 and MerTK expression. Moreover, by increasing the CD206 expression, the secretome increased the *C. albicans* phagocytosis activity of Mreg-polarized macrophages. According to our hypothesis, hBMSCs skew macrophages toward a proresolving phenotype that facilitate wound healing and restore homeostasis. Interestingly, DHA-hBMSCs also increased the expression of CD163 and decreased the gene expression of MMP-2 in Mreg-polarized macrophages indicating that the DHA modifications have an impact on the immunomodulatory properties of hBMSCs. These findings highlight the potential of PUFA supplementations as an easy and safe method to improve the hBMSC therapeutic potential.

## Figures and Tables

**Table 1:** Effect of hBMSC cell-cell contact and secretome with Mreg-polarized and Mreg-activated macrophages to cytokine production.

	Concentration, pg/mL (IQR)							
		Cell-Cell Contact	Cell-Cell Contact	Cell-Cell Contact	Secretome	Secretome	Secretome	
Cytokine	Mreg-Polarized	Mreg-Polarized +Control-hBMSC	Mreg-Polarized +DHA-hBMSC	Mreg-Polarized +AA-hBMSC	Mreg-Polarized +Control-hBMSC	Mreg-Polarized +DHA-hBMSC	Mreg-Polarized +AA-hBMSC	*p*-value ^a^
TNF-α	123.5 (131.5)	247.6 (199.7)	165.4 (278.8)	183.6 (177.2)	181.9 (434.9)	228.0 (273.8)	192.8 (350.3)	0.999
IL-10	39.5 (81.5)	48.0 (31.7)	36.9 (55.4)	36.1 (31.4)	32.5 (40.5)	29.1 (29.6)	53.6 (27.7)	0.975
IL-23	0.0 (0.0)	0.3 (0.9)	0.0 (0.0)	0.0 (0.0)	0.0 (0.0)	0.0 (0.0)	0.0 (0.0)	0.015
	**Mreg-activated**	**Mreg-activated +control-hBMSC**	**Mreg-activated +DHA-hBMSC**	**Mreg-activated +AA-hBMSC**	**Mreg-activated +control-hBMSC**	**Mreg-activated +DHA-hBMSC**	**Mreg-activated +AA-hBMSC**	***p*** **-value ^a^**
TNF-α	1591.6 (1898.5)	2106.7 (1825.5)	1887.1 (2041.1)	1899.1 (1672.3)	2025.5 (1512.2)	1950.5 (1895.9)	1823.6 (1959.4)	0.998
IL-10	31.0 (69.9)	45.5 (70.4)	39.6 (54.4)	33.9 (76.7)	31.5 (88.2)	45.0 (57.1)	33.3 (69.6)	0.871
IL-23	0.0 (0.1)	0.4 (2.7)	0.4 (1.0)	0.0 (0.2)	0.1 (0.2)	0.0 (0.4)	0.1 (0.4)	0.757

DHA, docosahexaenoic acid; AA, arachidonic acid; TNF, tumor necrosis factor; IL, interleukin; IQR, interquartile range. ^a^ The statistical significance of variation between groups was determined using the Kruskal-Wallis rank sum test.

## Supplementary Materials

The following are available online at https://www.mdpi.com/2073-4409/9/9/2142/s1, Figure S1: Gating strategy and CD90 exclusion, Figure S2: Gating strategy in imaging flow cytometry, Figure S3: The phagocytose assay results from the CD206 non-responders, Table S1: Effect of hBMSC cell-cell contact and secretome on the median fluorescence intensity of phenotype markers on Mreg-polarized and Mreg-activated macrophages, Table S2: Effect of hBMSC cell-cell contact and secretome on the frequency of positive cells of phenotype markers on Mreg-polarized and Mreg-activated macrophages.
