# Supplementary material for: Human Mesenchymal Stromal Cell Secretome Promotes the Immunoregulatory Phenotype and Phagocytosis Activity in Human Macrophages
Source: Cells. 2020 Sep 22;9(9):2142. doi: 10.3390/cells9092142 (PMC7564172; doi:10.3390/cells9092142)
Supplement: Supplementary file 1 [file cells-09-02142-s001.pdf]

SUPPLEMENTARY MATERIAL

Human mesenchymal stromal cell secretome promotes the immunoregulatory phenotype and phagocytosis activity in human macrophages

Minna Holopainen[1,2], Ulla Impola[1], Petri Lehenkari[3], Saara Laitinen[1]* & Erja Kerkelä[1]*

* Equal contribution

[1] Finnish Red Cross Blood Service, Helsinki, Finland
[2] Molecular and Integrative Biosciences Research Programme, Faculty of Biological and Environmental Sciences, University of Helsinki, Helsinki, Finland
[3] Department of Anatomy and Surgery, Institute of Translational Medicine, University of Oulu and Clinical Research Centre, Oulu, Finland

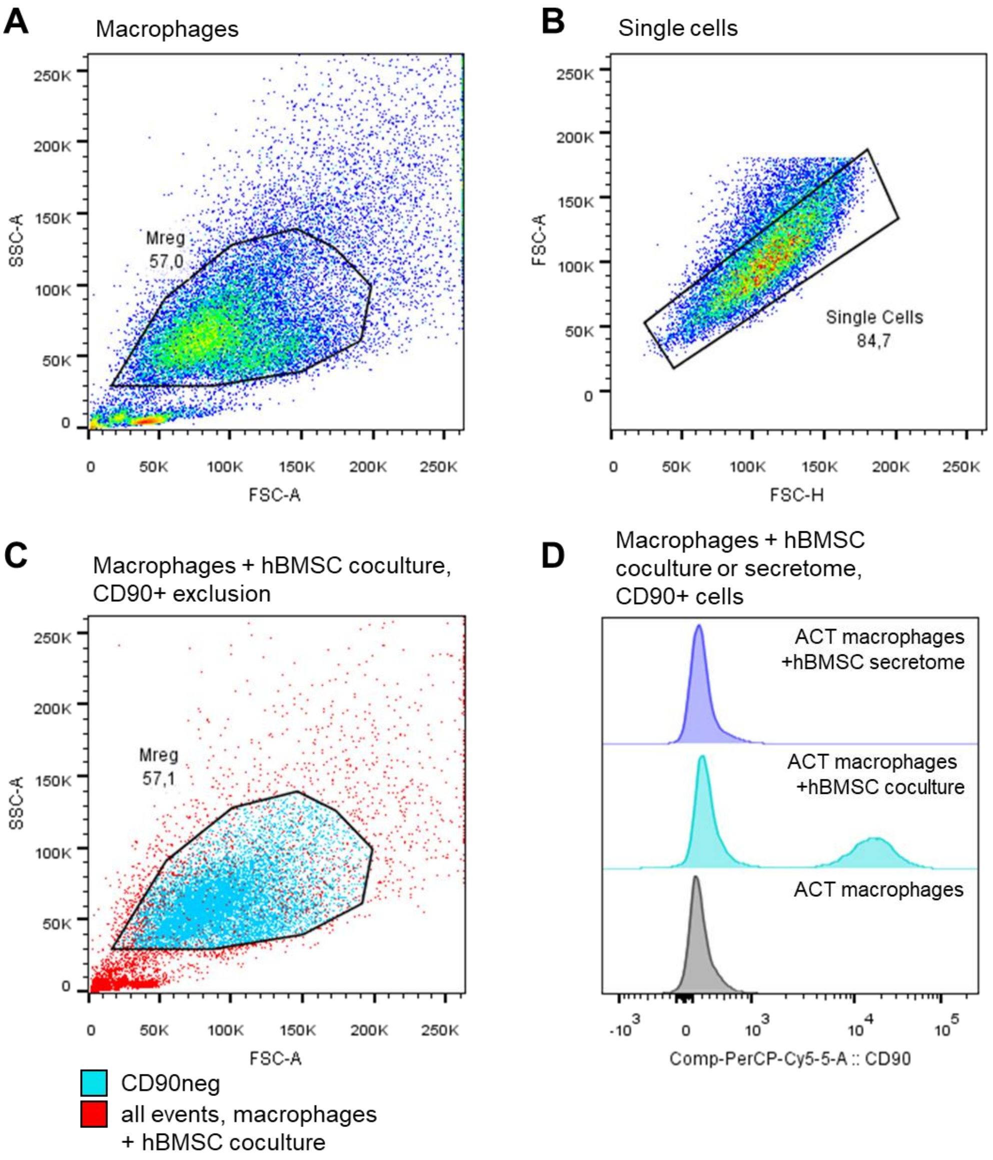

Figure S1. Gating strategy and CD90 exclusion. A representative figure of the gating strategy for the main macrophage population (A) and doublet discrimination (B). The CD90 positive hBMSCs were excluded from the analysis (C). Representative histograms demonstrate that CD90 positive cells were present only in the cell-cell contact setting (D). Mreg-activated, macrophages polarized and activated with 5 ng/mL M-CSF, 25 ng/mL IFN-γ and 10 ng/mL LPS.

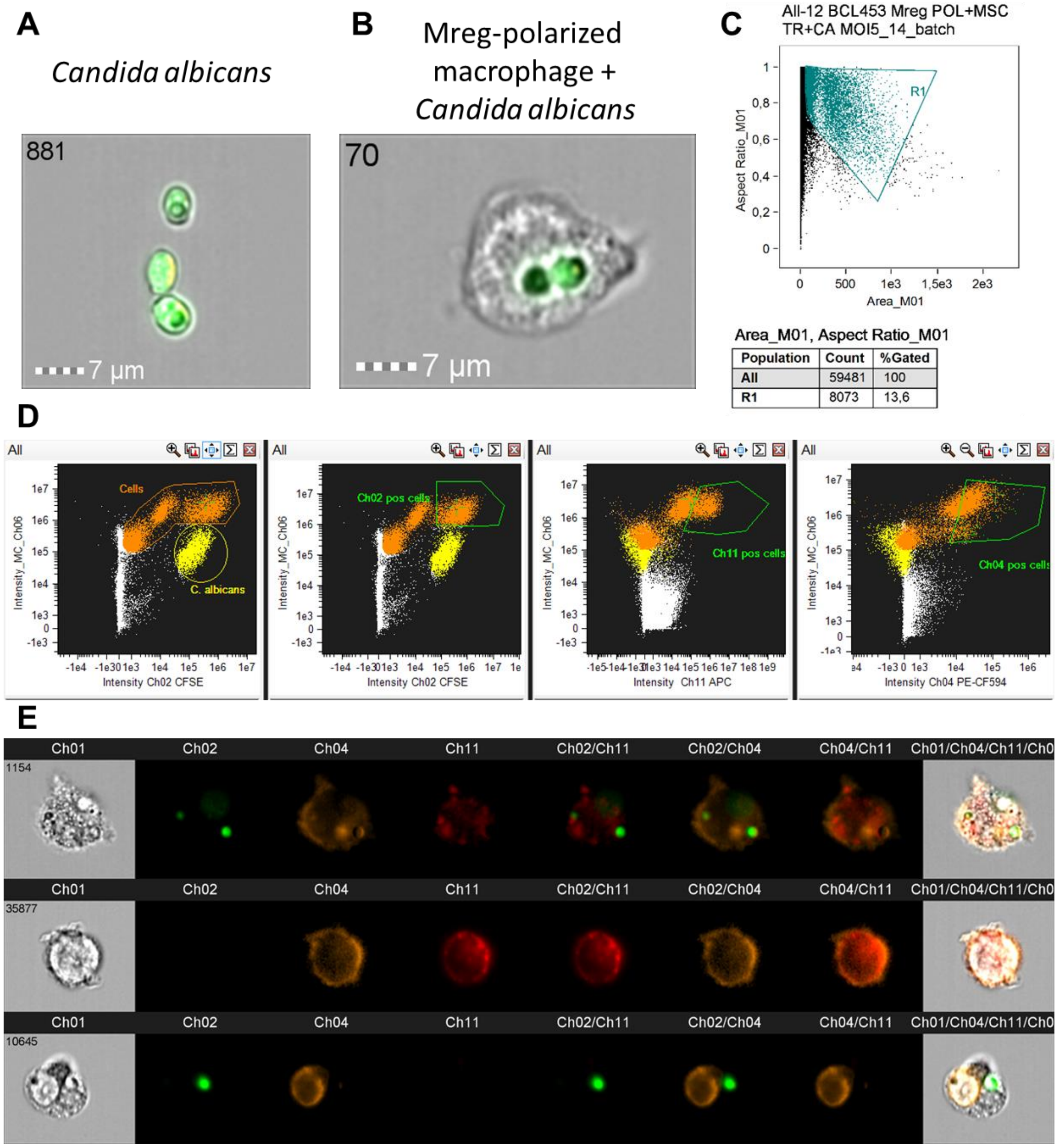

Figure S2. Gating strategy in imaging flow cytometry. A representative image of CFSE-stained Candida albicans (A) and Mreg-polarized macrophage that has phagocytosed CFSE-stained *C. albicans* (B). Representative images of the area aspect ratio (C) and the gating strategies (D) and stained macrophages (E). Ch01, bright field; Ch02, CFSE-dyed *C. albicans*; Ch04, CD86+ cells; Ch11, CD206+ cells.

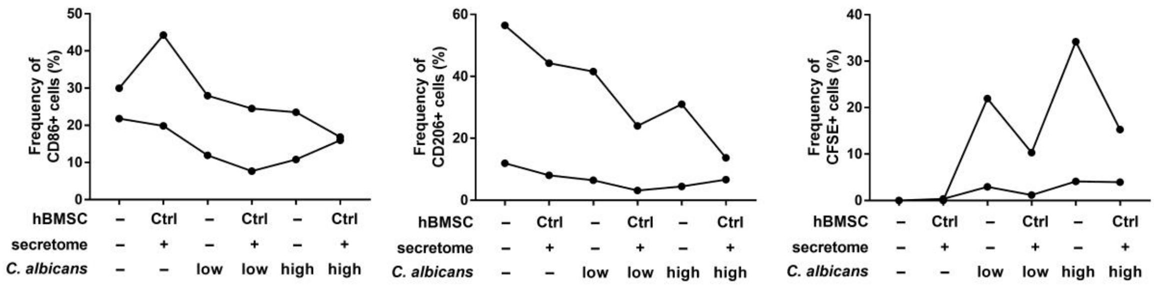

Figure S3. The phagocytose assay results from the CD206 non-responders. The frequency of CD86 and CD206 positive cells and the phagocytosis of CFSE-dyed *C. albicans* was determined with imaging flow cytometry. Non-responders were categorized by <1-fold change in CD206 expression, n = 2.

Table S1. Effect of hBMSC cell-cell contact and secretome on the median fluorescence intensity of phenotype markers on Mreg-polarized and Mreg-activated macrophages.

	Median fluorescence intensity (IQR)							
		Cell-cell contact	Cell-cell contact	Cell-cell contact	Secretome	Secretome	Secretome	
**Marker**	**Mreg polarized**	**Mreg polarized +control-hBMSC**	**Mreg polarized +DHA-hBMSC**	**Mreg polarized +AA-hBMSC**	**Mreg polarized +control-hBMSC**	**Mreg polarized +DHA-hBMSC**	**Mreg polarized +AA-hBMSC**	**p-value[a]**
CD86	4225.5 (1832.5)	4401.0 (1738.8)	3267.0 (1026.5)	3987.0 (1602.3)	4830.5 (1759.5)	4880.0 (2290.5)	4655.0 (2531.8)	0.603
HLA-DR	7066.0 (2595.3)	5433.5 (3312.5)	5439.0 (656.5)	5701.0 (2112.0)	8255.0 (2787.0)	8206.0 (2789.3)	7295.0 (3680.3)	0.0849
CD206	207.5 (121.8)	216.0 (45.8)	204.5 (29.8)	203.0 (48.5)	540.0 (529.5)	587.5 (504.5)	467.5 (286.5)	< 0.001
CD163	485.0 (47.5)	481.0 (106.0)	438.0 (63.5)	443.0 (47.5)	508.0 (1285.0)	597.5 (1370.5)	529.5 (151.5)	0.0631
PD-L1	743.5 (304.3)	693.0 (505.8)	742.5 (208.8)	701.0 (383.5)	897.5 (286.0)	952.0 (317.5)	804.0 (248.0)	0.519
TNFR2	701.5 (2060.5)	732.5 (1903.0)	727.5 (1478.0)	741.5 (1378.8)	776.5 (1180.0)	728.0 (1596.8)	819.0 (1348.0)	0.994
MerTK	2623.5 (986.0)	2184.0 (937.3)	2160.0 (141.8)	2268.5 (434.8)	3036.5 (1013.0)	3015.0 (1026.8)	2783.5 (1280.5)	0.158
	**Mreg activated**	**Mreg activated +control-hBMSC**	**Mreg activated +DHA-hBMSC**	**Mreg activated +AA-hBMSC**	**Mreg activated +control-hBMSC**	**Mreg activated +DHA-hBMSC**	**Mreg activated +AA-hBMSC**	**p-value[a]**
CD86	8716.5 (8141.3)	10004.0 (9860.5)	11150.0 (8725.0)	10079.5 (7553.5)	14305.5 (13777.5)	14384.0 (10645.8)	16368.0 (14422.8)	0.527
HLA-DR	9594.5 (7650.3)	7445.5 (8869.5)	10059.0 (8446.3)	8166.0 (7598.3)	12924.0 (12574.5)	13767.5 (8951.3)	14743.5 (11476.0)	0.412
CD206	204.0 (112.5)	189.5 (119.0)	195.0 (95.3)	188.5 (113.0)	463.5 (534.5)	684.5 (282.3)	663.0 (292.5)	< 0.001
CD163	461.5 (130.8)	428.5 (184.3)	448.5 (174.8)	381.0 (124.3)	551.0 (92.8)	633.5 (654.5)	558.0 (245.3)	0.002
PD-L1	896.5 (562.8)	744.0 (579.3)	893.5 (647.5)	756.0 (473.5)	1019.5 (833.5)	1123.0 (616.8)	1089.0 (685.3)	0.911
TNFR2	826.5 (2826.5)	1019.5 (3142.0)	1117.5 (3132.0)	1027.5 (2938.3)	1386.0 (3662.0)	1302.5 (2963.0)	1376.0 (2872.0)	0.907
MerTK	3985.0 (3417.0)	3524.0 (3776.5)	4445.5 (3547.3)	3740.5 (3147.3)	5632.0 (5350.8)	5920.0 (3880.3)	6428.5 (5212.5)	0.432

Mreg-polarized, macrophages polarized with 5 ng/mL M-CSF; Mreg-activated, macrophages polarized and activated with 5 ng/mL M-CSF, 25 ng/mL IFN-γ and 10 ng/mL LPS; hBMSC, human bone marrow derived mesenchymal stromal cell; DHA, docosahexaenoic acid; AA, arachidonic acid; IQR, interquartile range.

[a] The statistical significance of variation between groups was determined using the Kruskal-Wallis rank sum test.

Table S2. Effect of hBMSC cell-cell contact and secretome on the frequency of positive cells of phenotype markers on Mreg-polarized and Mreg-activated macrophages.

		Median frequency of positive cells, % (IQR)						
		Cell-cell contact	Cell-cell contact	Cell-cell contact	Secretome	Secretome	Secretome	
**Marker**	**Mreg polarized**	**Mreg polarized +control-hBMSC**	**Mreg polarized +DHA-hBMSC**	**Mreg polarized +AA-hBMSC**	**Mreg polarized +control-hBMSC**	**Mreg polarized +DHA-hBMSC**	**Mreg polarized +AA-hBMSC**	**p-value[a]**
CD86	78.0 (16.2)	87.8 (13.0)	81.0 (20.2)	85.7 (11.4)	86.0 (9.8)	88.4 (12.2)	87.0 (19.9)	0.914
HLA-DR	99.7 (0.6)	99.4 (0.5)	99.4 (0.6)	99.4 (0.4)	99.9 (0.2)	99.8 (0.4)	99.9 (0.3)	0.054
CD206	34.6 (30.6)	24.9 (14.3)	22.6 (6.1)	23.3 (8.5)	62.7 (15.8)	63.0 (23.4)	54.7 (18.3)	< 0.001
CD163	5.5 (19.1)	0.6 (14.4)	0.5 (10.2)	0.9 (3.5)	0.3 (43.3)	0.3 (42.0)	1.2 (29.6)	0.996
PD-L1	59.2 (21.6)	88.6 (36.9)	76.5 (29.3)	82.6 (22.0)	90.4 (41.8)	87.9 (46.9)	83.4 (36.9)	0.965
TNFR2	0.2 (0.2)	0.1 (0.2)	0.1 (0.3)	0.1 (0.2)	0.6 (0.6)	0.3 (0.2)	0.3 (0.3)	0.082
MerTK	0.2 (0.9)	0.1 (0.1)	0.3 (0.6)	0.2 (0.4)	0.3 (0.5)	0.4 (0.6)	0.1 (0.4)	0.917
	**Mreg activated**	**Mreg activated +control-hBMSC**	**Mreg activated +DHA-hBMSC**	**Mreg activated +AA-hBMSC**	**Mreg activated +control-hBMSC**	**Mreg activated +DHA-hBMSC**	**Mreg activated +AA-hBMSC**	**p-value[a]**
CD86	97.1 (6.5)	91.1 (12.6)	89.8 (9.6)	89.3 (11.7)	97.3 (9.6)	97.1 (4.0)	97.6 (3.4)	0.392
HLA-DR	99.9 (0.1)	98.4 (1.0)	98.6 (1.1)	98.6 (1.5)	99.9 (0.7)	99.9 (0.1)	99.8 (0.1)	< 0.001
CD206	22.5 (7.0)	10.9 (9.1)	12.8 (5.8)	12.9 (6.0)	54.9 (15.2)	65.0 (9.0)	67.1 (6.3)	< 0.001
CD163	0.5 (2.5)	0.1 (1.1)	0.1 (1.1)	0.1 (1.1)	0.3 (30.5)	0.3 (39.4)	0.4 (32.8)	0.897
PD-L1	91.5 (2.7)	77.0 (21.9)	78.2 (9.8)	75.1 (6.3)	86.4 (40.2)	90.6 (20.7)	92.6 (29.0)	0.382
TNFR2	0.0 (0.0)	0.1 (0.1)	0.0 (0.3)	0.1 (0.4)	1.0 (1.2)	0.9 (1.5)	0.6 (1.0)	0.062
MerTK	0.3 (0.4)	1.2 (2.0)	1.8 (1.5)	0.9 (3.2)	8.1 (8.6)	6.5 (9.1)	5.7 (5.9)	0.032

Mreg-polarized, macrophages polarized with 5 ng/mL M-CSF; Mreg-activated, macrophages polarized and activated with 5 ng/mL M-CSF, 25 ng/mL IFN-γ and 10 ng/mL LPS; hBMSC, human bone marrow derived mesenchymal stromal cell; DHA, docosahexaenoic acid; AA, arachidonic acid; IQR, interquartile range.

[a] The statistical significance of variation between groups was determined using the Kruskal-Wallis rank sum test.
